# IgA Nephropathy in a Patient Presenting with Pseudotumor Cerebri

**DOI:** 10.1155/2016/5273207

**Published:** 2016-02-16

**Authors:** Umair Syed Ahmed, Patrick Bacaj, Hafiz Imran Iqbal, Songul Onder

**Affiliations:** ^1^Section of Nephrology, Department of Medicine, West Virginia University, Morgantown, WV 26506, USA; ^2^Department of Pathology, West Virginia University, Morgantown, WV 26506, USA

## Abstract

IgA nephropathy is the most common glomerulonephritis worldwide and typically has minimal signs for chronicity in histopathology at the time of initial presentation. Pseudotumor cerebri (PTC) is characterized by increased intracranial pressure in the absence of any intracranial lesions, inflammation, or obstruction. PTC has been reported in renal transplant and dialysis patients, but we are unaware of any reports of pseudotumor cerebri in patients with IgA nephropathy. We report a case of a young female who presented with signs and symptoms of pseudotumor cerebri and was subsequently diagnosed with IgA nephropathy and end-stage renal disease. To our knowledge this is the first report of IgA nephropathy presenting as end-stage renal disease in a patient who presented with pseudotumor cerebri.

## 1. Introduction

IgA nephropathy is the most prevalent primary glomerular disorder in the world [1–3], first described by Berger and Hinglais in 1968 [4]. It is a mesangial proliferative glomerulonephritis, characterized by diffuse mesangial IgA deposition. Clinical features range from asymptomatic hematuria to rapidly progressive glomerulonephritis (RPGN). It is most often associated with microscopic or recurrent macroscopic hematuria, proteinuria, and chronic kidney disease. Although it is a benign disease in most patients, chronic kidney disease and end-stage renal disease (ESRD) occur in about 20–40% of patients within decades of presentation. While IgA nephropathy typically involves only the kidneys, it has been reported in patients with liver cirrhosis [5], celiac disease [6], rheumatoid arthritis [7], and ankylosing spondylitis [8].

Idiopathic intracranial hypertension (IIH) or pseudotumor cerebri (PTC) is a neurological disorder that is also known as benign intracranial hypertension (BIH). It is characterized by markedly elevated intracranial pressures in the absence of an intracranial lesion, inflammation, or obstruction. PTC has been reported in association with kidney disease [9], including kidney transplantation [10], and patients on dialysis [11]. Despite its name, pseudotumor cerebri is not always benign and can be associated with debilitating symptoms including severe headaches and permanent visual loss. Therefore, the terms idiopathic intracranial hypertension and pseudotumor cerebri are more accurate than “benign” intracranial hypertension [12].

Pseudotumor cerebri most commonly occurs in obese women of childbearing age but may be seen in children, men, and older adults. The presentation is characterized by elevated cerebrospinal fluid (CSF) pressures of more than 250 mm H_2_O, along with symptoms and signs of elevated intracranial pressures. Symptoms include headaches, visual loss, and pulsatile tinnitus, while signs include papilledema, visual field defects, and sixth nerve palsy.

We present a case of a young female who presented with signs and symptoms of pseudotumor cerebri and was subsequently diagnosed with IgA nephropathy with ESRD.

## 2. Case

Patient is a 31-year-old Caucasian female, with a BMI of 33 and no known comorbid conditions, who presented to her ophthalmologist with a few-month history of headaches and blurred vision. The patient was not on any medications, including oral contraceptives. Her fundoscopic eye exam revealed papilledema. Patient was subsequently admitted to an outside hospital for an evaluation of any possible intracranial lesions which could potentially be resulting in papilledema.

On admission at the outside hospital, blood pressure was 150/70 mm Hg. Serum creatinine level was 3.7 mg/dL on admission. Due to lack of previous medical evaluation, patient's baseline serum creatinine was not known. Computerized tomography scan of the brain without contrast was unremarkable. Magnetic resonance imaging of the brain done subsequently showed minimal white matter changes, raising concern for posterior reversible encephalopathy syndrome. A lumbar puncture done revealed an elevated opening pressure of 460 mm H_2_O. CSF analysis was unremarkable. With clinical concerns for pseudotumor cerebri, patient was started on Furosemide and Acetazolamide. Hydralazine was started due to elevated blood pressure which subsequently improved. Over the course of her stay, patient's renal parameters worsened. An ultrasound of the kidneys was unremarkable, with normal kidney size, cortical thickness, and echogenicity. Duplex ultrasonography of the renal artery reportedly showed beading, raising concern for fibromuscular dysplasia. The patient was then transferred to WVU Hospital for further evaluation.

On arrival at our hospital, serum creatinine was 5.15 mg/dL. Microscopic urine analysis showed 5 red blood cells per high power field, with an unremarkable urine sediment. A random urine protein/creatinine ratio showed 2.3 grams of proteinuria. A CO_2_ angiogram was unremarkable for any renal artery stenosis or microaneurysms. Repeat renal artery duplex ultrasonography at our institution did not show renal artery stenosis. CT angiogram or renal arteriogram was not pursued due to renal insufficiency. Both serum protein electrophoresis and serum-free light chain assay were unremarkable. Viral serologies and serum markers of vasculitis were also negative.

**Figure 1:**
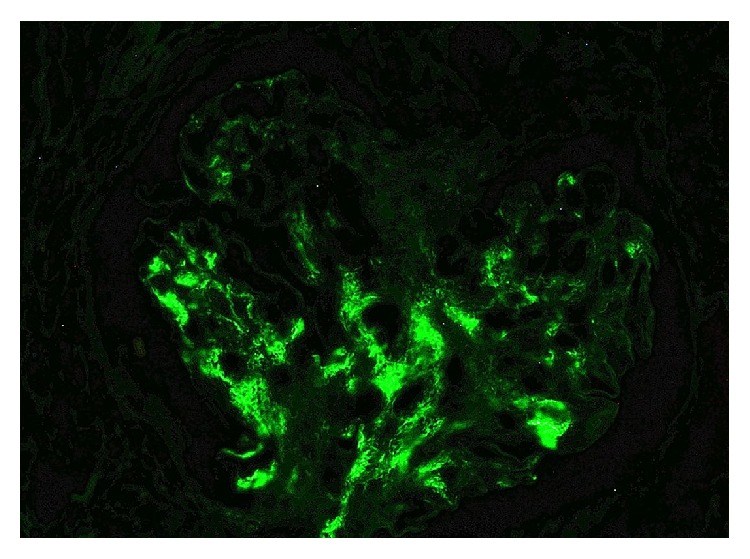
Immunofluorescence demonstrating positivity to IgA.

**Figure 2:**
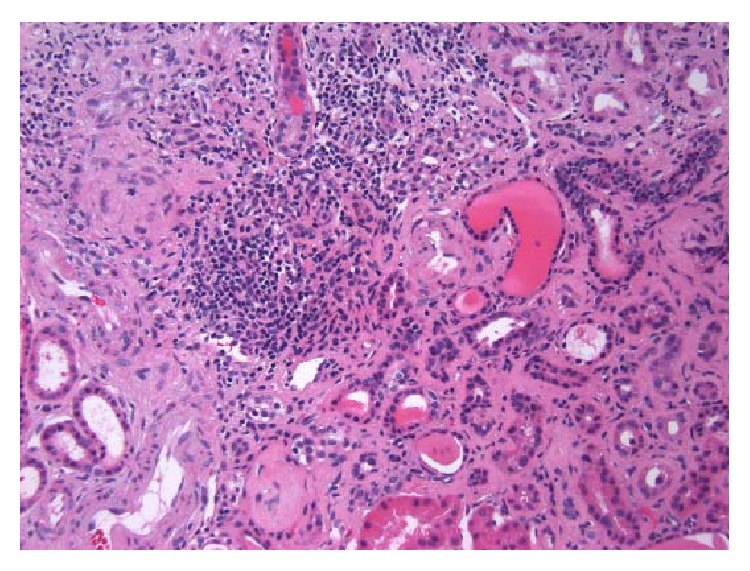
Interstitial fibrosis and chronic inflammation (H&E, 200x).

**Figure 3:**
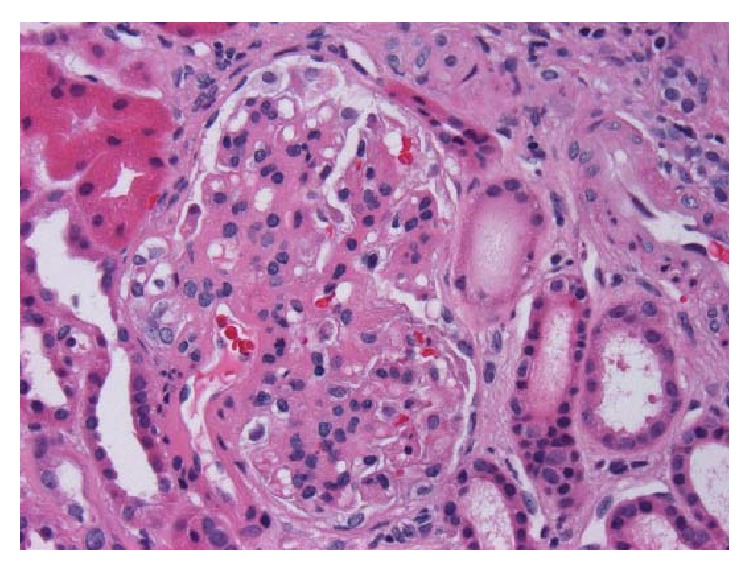
Glomerulus with thickened mesangium and segmental mesangial hypercellularity (H&E, 400x).

**Figure 4:**
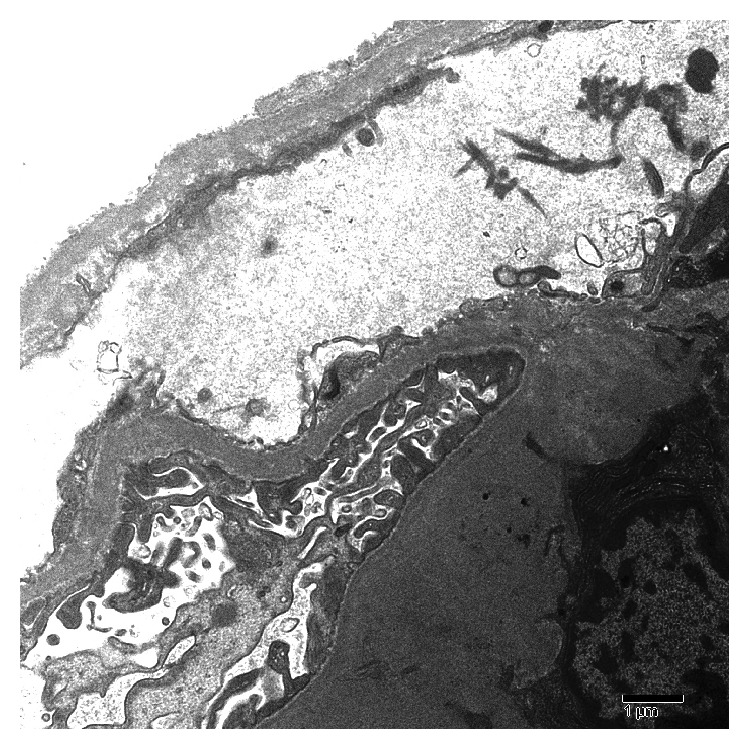
Large mesangial deposit of amorphous electron-dense material (EM).

Patient was started on oral sodium bicarbonate supplements for a high anion gap metabolic acidosis, which was assessed to be due to renal insufficiency rather than the use of Acetazolamide. Due to persistent visual symptoms, patient underwent surgery to place a ventriculoperitoneal shunt to relieve excess CSF pressure. Visual symptoms improved after placement of the shunt. Patient's renal parameters continued to deteriorate during her hospital stay and she started developing symptoms of uremia. She underwent an ultrasound guided kidney biopsy which showed IgA nephropathy (Figure 1) with moderate interstitial fibrosis and global glomerulosclerosis involving 75% of glomeruli, indicating advanced chronic kidney disease (Figures 2, 3, and 4). Patient was subsequently started on hemodialysis via a tunneled dialysis catheter.

## 3. Discussion

Pseudotumor cerebri is a diagnosis of exclusion and therefore other causes of elevated intracranial pressure such as malignancy, abscesses, hydrocephalus, meningoencephalitis, and intracranial hemorrhage need to be ruled out. Our patient had an extensive evaluation, including CT and MRI of the brain and a CSF analysis, which were all unremarkable.

The pathogenesis of pseudotumor cerebri is unknown. The postulated mechanisms include cerebral venous outflow abnormalities (such as venous stenosis and venous hypertension); increased cerebrospinal fluid (CSF) outflow resistance at either the level of the arachnoid granulations or CSF lymphatic drainage sites; obesity-related increased abdominal and intracranial venous pressure; altered sodium and water retention mechanisms; and abnormalities of vitamin A metabolism [13, 14]. Pseudotumor cerebri has been reported in patients using tetracyclines [15], lithium [16], high dose vitamin A [17], oral contraceptives [18], growth hormone therapy [19], nitrofurantoin [20], and nalidixic acid [21]. Our patient was not on any medications prior to her hospital admission.

While there are numerous conditions and medications which have been associated with pseudotumor cerebri, case reports of its occurrence in patients with kidney disease are limited. It has been reported in kidney transplant recipients [10, 22], who are thought to be at an increased risk due to the use of corticosteroids, the presence of anemia, a hypercoagulable state, and a weight gain [23, 24]. The majority of these patients were on cyclosporine A [25–28]. Its clinical manifestations improved/resolved on discontinuation of the medication. While pseudotumor cerebri has been seen in patients with bone marrow transplantation, its occurrence in renal transplant patients is not common.

Chang et al. [9] reported the occurrence of pseudotumor cerebri in a young male with chronic renal failure of unknown etiology. His headaches persisted despite repeated lumbar punctures and resolved after dialysis was started. This raises the question of whether uremia and pseudotumor cerebri are related, even though it is unknown what the underlying pathophysiology could be. Animal studies and pathology studies have shown that severe renal insufficiency could be associated with slightly elevated intracranial pressures [27–30]. This could be secondary to fluid overload, anemia, and increased cerebral blood flow. Interestingly intracranial pressures have been noted to be high during both hemodialysis [31] and peritoneal dialysis [11], as well as after dialysis, if patients developed dialysis disequilibrium syndrome [32].

Pseudotumor cerebri as a presentation of IgA nephropathy has not been reported in the medical literature. We report a case of a young obese female with PTC, IgA nephropathy, and advanced kidney disease. The clinical significance of this interesting observation is uncertain. Our patient was a young obese female and would therefore have been at relatively high risk of developing pseudotumor cerebri. The associated findings of IgA nephropathy and renal failure may have been coincidental. Alternatively, PTC may be a rare clinical manifestation of IgA nephropathy, particularly when it is complicated by uremia.
